# Multimode optical fiber transmission with a deep learning network

**DOI:** 10.1038/s41377-018-0074-1

**Published:** 2018-10-03

**Authors:** Babak Rahmani, Damien Loterie, Georgia Konstantinou, Demetri Psaltis, Christophe Moser

**Affiliations:** 1 Ecole Polytechnique Fédérale de Lausanne, Laboratory of Applied Photonics Devices, CH-1015 Lausanne, Switzerland; 2 Ecole Polytechnique Fédérale de Lausanne, Laboratory of Optics, CH-1015 Lausanne, Switzerland

## Abstract

Multimode fibers (MMFs) are an example of a highly scattering medium, which scramble the coherent light propagating within them to produce seemingly random patterns. Thus, for applications such as imaging and image projection through an MMF, careful measurements of the relationship between the inputs and outputs of the fiber are required. We show, as a proof of concept, that a deep neural network can learn the input-output relationship in a 0.75 m long MMF. Specifically, we demonstrate that a deep convolutional neural network (CNN) can learn the nonlinear relationships between the amplitude of the speckle pattern (phase information lost) obtained at the output of the fiber and the phase or the amplitude at the input of the fiber. Effectively, the network performs a nonlinear inversion task. We obtained image fidelities (correlations) as high as ~98% for reconstruction and ~94% for image projection in the MMF compared with the image recovered using the full knowledge of the system transmission characterized with the complex measured matrix. We further show that the network can be trained for transfer learning, i.e., it can transmit images through the MMF, which belongs to another class not used for training/testing.

## Multimode fibres: Neural network descrambling

A convolutional neural network (CNN) can successfully learn the nonlinear transmission characteristics of a multimode fibre thus allowing accurate image transmission and reconstruction. Propagation along a multimode fibre usually scrambles an input image, resulting in a seemingly random speckle pattern at the output. Babak Rahmani and coworkers from the Ecole Polytechnique Fédérale de Lausanne in Switzerland have now shown that a deep neural network(either a 22-layer CNN based on VGG-net technology or a 20-layer CNN based on Res-net technology) can learn the input-output relationship in a 0.75 m long piece of multimode fibre) and thus undo this scrambling. Experiments showed that both neural networks could perform highly accurate image reconstruction with an image fidelity as high as ~98% for image reconstruction and ~94% for image projection in the best case.

## Introduction

Multimode fibers (MMF) scramble the waves propagating inside them and produce seemingly random patterns known as speckles at their outputs. Despite this seemingly random nature, the system consisting of an input pattern, propagating through an MMF and a detector, behaves deterministically. It has been shown by many studies that image transmission or imaging through an MMF could be carried out, for example, by analog phase conjugation^1–5^, digital iterative methods^6–10^, digital phase conjugation^11,12^ or measuring experimentally the amplitude and phase of the output patterns corresponding to each input pattern to construct a matrix of complex numbers relaying the input to the output^13–16^. In the latter, the experiment requires an external reference beam brought to the output of the fiber to generate an interference pattern from which the complex optical field (amplitude and phase) can be extracted. Careful phase tracking needs to be implemented to correct for phase drift, which further complicates the implementation. Although some work has shown that the reference beam can also be sent through the same MMF^17^, multiple quadrature phase measurements must be done to extract the phase. A system in which a camera is placed at the output side of the fiber, which detects only the intensity of the output beam, is much simpler to implement. Some recent work used convex optimization to infer the matrix from the intensity measurement only^18,19^.

With intensity-only detection, the optical system is nonlinear and consists of finding what input phase (or amplitude) generates the intensity pattern detected at the output. We note that when a phase pattern *ϕ*(*x*, *y*) is used at the input, the system has two nonlinearities: the first is the conversion from the phase pattern *ϕ*(*x*, *y*) to the actual phase pattern displayed on the spatial light phase modulator *e*^*iϕ*(*x*, *y*)^ and the second is the square law of the detector, which takes the modulus of the output complex optical field $\left| {E\left( {x,y} \right)} \right|^2$. We explore, in this paper, whether neural networks are able to learn this nonlinear output to input a relationship without any a priori knowledge of the light propagation in the MMF system.

The idea of using neural networks in conjunction with MMFs has been around for almost three decades^20–22^. In Ref. ^20^, a neural network with a three-layer perceptron structure was used to classify 10 categories of images transmitted through the fiber. This simple neural network could not recognize images, for which it was not trained. In another experiment with an MMF, a single hidden layer neural network was utilized to classify a speckle pattern corresponding to an input code for the purpose of increasing the transmission capacity of the fiber optic system^21,22^.

Today, thanks to the ubiquitous availability of processing power via graphical processing units and new types of neural network architectures, a revival of applications using neural networks is happening and summarized in Ref. ^23^. Convolutional neural networks (CNNs) are a subclass of neural networks, which have been proposed to surpass the performance of other neural networks by decreasing the computation cost of fully connected layers through parameter sharing and use of sparse filters while, at the same time, increasing the number of layers in the network to achieve deep networks for solving more complex problems and speeding up the computations. With this new computational power, CNNs have been recently applied to imaging systems^24^. For example, in microscopy, deep CNNs have been successfully used to provide resolution enhancement in images of a same class of histology samples^25^ and phase recovery in nonlinear inverse problems^26,27^, i.e., obtaining the phase at one plane when the intensity is measured at another imaging plane. In other words, the neural network is able to learn the Fresnel propagation kernel and the intensity square law.

In this work, we propose to investigate whether CNNs can be applied to learn the propagation of light in an MMF when only intensity detection is performed at its output.

In the first part, we demonstrate that a deep CNN is able to learn two types of *nonlinear* inverse problems: 1-amplitude-to-amplitude and 2- amplitude-to-phase. As indicated above, the former nonlinearity is due to the detector square law and the latter adds another nonlinearity due to the complex exponential dependence of the phase at the input of the fiber. The CNN is trained with pairs of images, which are the fiber output amplitude speckles corresponding to the spatial light modulator (SLM) input phases (amplitude) associated with the database of handwritten Latin alphabet. This experiment corresponds to an amplitude-to phase/amplitude mapping. We note that this choice of database is not unique. We particularly use two types of network architectures for this task. One is a 22-layer CNN, which uses VGG-nets^28^ style, while the other is a 20-layer CNN based on residual networks^29–31^ (Res-net). We show that the former can generate input SLM amplitude and phase patterns with average two-dimensional correlations of ~93% for amplitude patterns and ~79% for phase patterns (and can reach fidelities as high as ~98% and ~85%, respectively) for the validation set, while the latter architecture reproduces input amplitudes with a fidelity of ~96% and input phases with a fidelity of ~88% with a much faster convergence rate.

In the second part, we show that the network is able to reconstruct images that have not been seen by the network, i.e., which are not in the training or validation set. Specifically, we show that images belonging to a different class (handwritten digits, a heart picture, a house, etc.) can be reconstructed with an ~90% fidelity through the MMF. This result shows that the trained CNN is able to do transfer learning in the MMF system. To our knowledge, this has not been demonstrated before in MMFs.

Finally, we demonstrate the ability of CNNs to project arbitrary patterns through the MMF using neural networks. For this, we use the measured transmission matrix and compute its inverse to generate examples of SLM phase patterns that produce the amplitude images of the database of handwritten alphabets at the output. The CNN is then trained with this set. The phase patterns generated by the network are then displayed on the SLM experimentally and we verified that the desired intensity patterns were generated at the output of the MMF with a fidelity that could be as high as ~94%. This result is interesting as the trained CNN is effectively replicating the capability of the matrix. We note that the measured matrix fully describes the transmission because the system is linear in the complex optical field. However, to access it, nontrivial interferometric approaches are needed. In contrast, the proposed neural network only uses the amplitude of the output pattern to deduce the input phase/amplitude field, which considerably reduces the implementation complexity for learning light transmission in MMFs.

## Results

### Amplitude-to-amplitude inversion

**Fig. 1:**
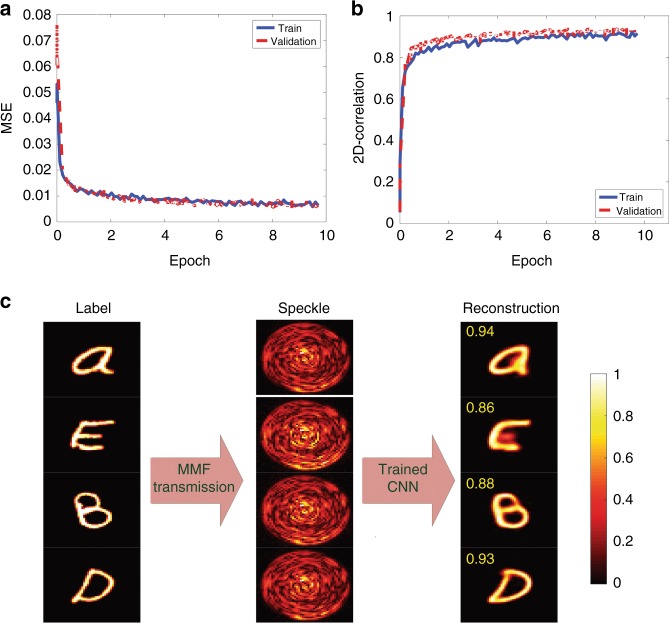
Amplitude-to-amplitude inversion. Performance of the network in reconstructing the input amplitudes from the output amplitude speckle patterns when the CNN is trained with the handwritten Latin alphabet. The speckle pattern for each letter image is obtained using the transmission matrix of the system. Calculated (**a**) MSE and **b** 2D-correlation for the train/validation datasets. **c** Examples of the output amplitude speckle patterns and the reconstructed fiber input amplitude patterns produced via the CNN. The fidelity number for each reconstructed image with respect to its corresponding grayscale label is shown

In the first part, the inverse problem of regenerating the input amplitudes from the output amplitudes in an MMF is investigated. We train both CNNs (VGG-net and Res-net) with 60000 image pairs and test on the 1000 images in the validation dataset. Each pair contains an image of the Latin alphabet adopted from Ref. ^32^, together with the corresponding output speckle amplitude obtained at the distal end of the fiber (see data preparation subsection in “Materials and Methods” section). Figure 1 plots the mean squared error (MSE) as well as the 2D-correlation between the labels and the reconstructed SLM amplitude patterns belonging to the Latin alphabet when the amplitude input image is used (Fig. 1a, b) to train the VGG-net CNN. Examples of the labels, corresponding speckles and reconstructed amplitude patterns in the validation dataset are shown in Fig. 1c.

Similarly, Supplementary Fig. S9 plots the 2D-correlation when the Res-net style CNN is used for training with the same data set. Table 1 quantitatively compares the performance of the two CNNs for this task.

**Table 1:** Comparison of the VGG-net and Res-net performance on the amplitude-to-amplitude/amplitude-to-phase inversions for the validation data set

Network Architecture	2D correlation at convergence		Training time	
	Amplitude-to-amplitude	Amplitude-to-phase	Amplitude-to-amplitude	Amplitude-to-phase
VGG-net	~0.93	~0.79	~4 h 35 min	~9 h 0 min
Res-net	~0.96	~0.88	~1 h 20 min	~1 h 0 min

### Amplitude-to-phase inversion

**Fig. 2:**
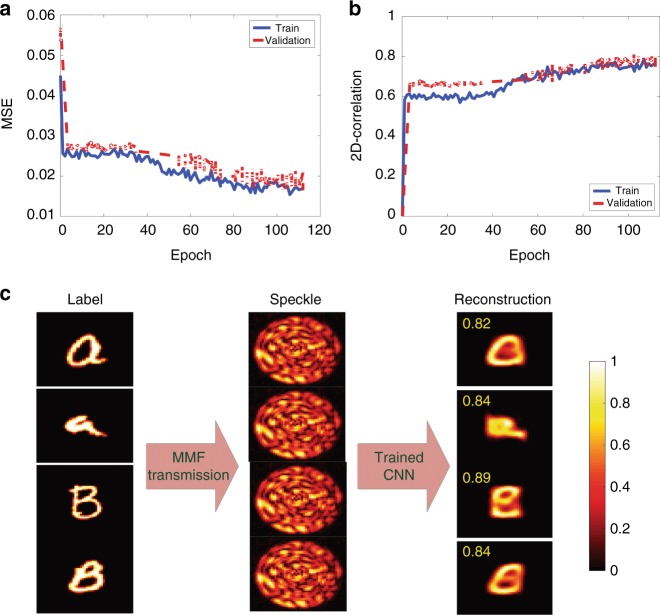
Amplitude-to-phase inversion. Performance of the network in reconstructing the input phases from the output amplitude speckle patterns when the CNN is trained with the handwritten Latin alphabet. The speckle pattern for each letter image is obtained using the transmission matrix of the system. Calculated (**a**) MSE and (**b**) 2D-correlation for the train/validation data sets. **c** Examples of the output amplitude speckle patterns and the reconstructed fiber input phase patterns produced via the CNN. The fidelity number for each reconstructed image with respect to its corresponding grayscale label is shown

In the second part, we study the amplitude-to-phase inversion on the same type of images, i.e., the Latin alphabet. Therefore, we train the CNN with phase modulated images of the Latin alphabet. Figure 2 plots the MSE as well as the 2D-correlation between the labels and the reconstructed SLM phase patterns belonging to the Latin alphabet when the phase input image is used (Fig. 2a, b). Examples of the labels, corresponding speckles and reconstructed amplitude patterns in the validation dataset are shown in Fig. 2c (also refer to Table 1 and Supplementary Fig. S9 for a comparison between the results of VGG-net and Res-net).

### Transfer learning

We show in the previous section that both CNNs are able to perform the inverse mapping between the amplitude at the distal end and the phase/amplitude at the proximal end of the fiber. An interesting question is whether the network is able to transfer its learning to other images, i.e., images belonging to other categories that have never been seen by the network even in the training step.

**Fig. 3:**
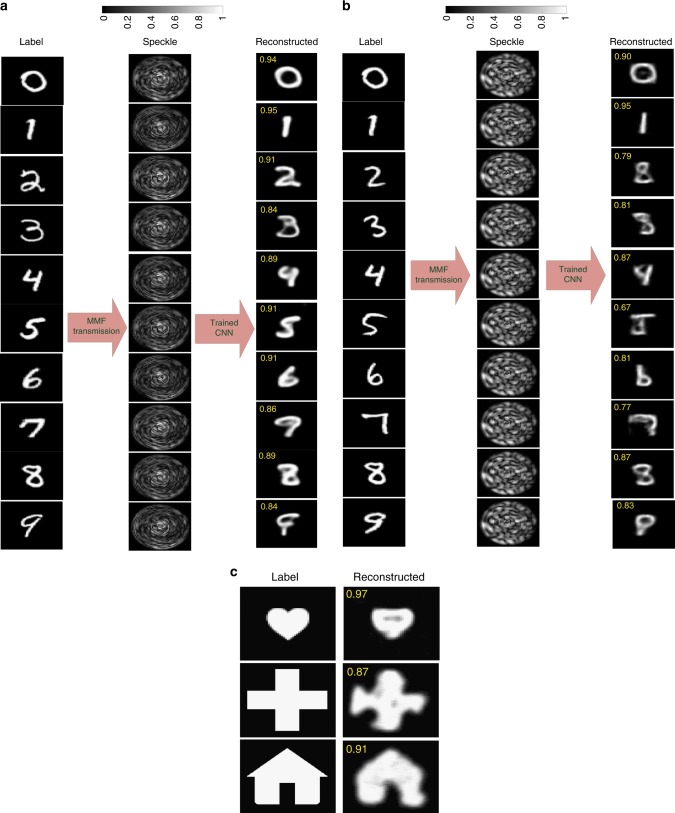
Transfer learning. Performance of the network in transfer learning the reconstruction of the input amplitudes/phases from the output amplitude speckle patterns when the CNN is trained with the handwritten Latin alphabet. The speckle pattern for each image is obtained using the transmission matrix of the system. **a** Reconstructed amplitude input patterns and **b** reconstructed phase input patterns of digit images. **c** Example of reconstructed amplitude input patterns for other images. The fidelity for each reconstructed image with respect to its corresponding label is shown

We use the VGG-net CNN that was trained on the amplitude of the speckle patterns and the input amplitude/phase of the Latin alphabet dataset. We use images of the digit dataset^32^ for evaluating the transfer learning reconstruction performance. The dataset for training/testing is thus not of the same class of images, although they are both grayscale and of a handwritten nature. Examples of (a) the reconstructed amplitude input pattern and (b) the reconstructed phase input pattern by the CNN belonging to another class are depicted in Fig. 3a, b, respectively. The reconstructed amplitude input pattern for a heart picture, as well as other images, is also shown in Fig. 3c, which shows a good visual fidelity (~90% correlation with the labels). This demonstrates the ability of the network to perform transfer learning in the MMF system.

Additionally, Supplementary Movie 1 and 2 contain animations of a “Moving Donuts” as well as a “Running Dog”, which are obtained by the network to further illustrate transfer leaning (See the Supplementary Materials).

### Experimental validation and image projection

**Fig. 4:**
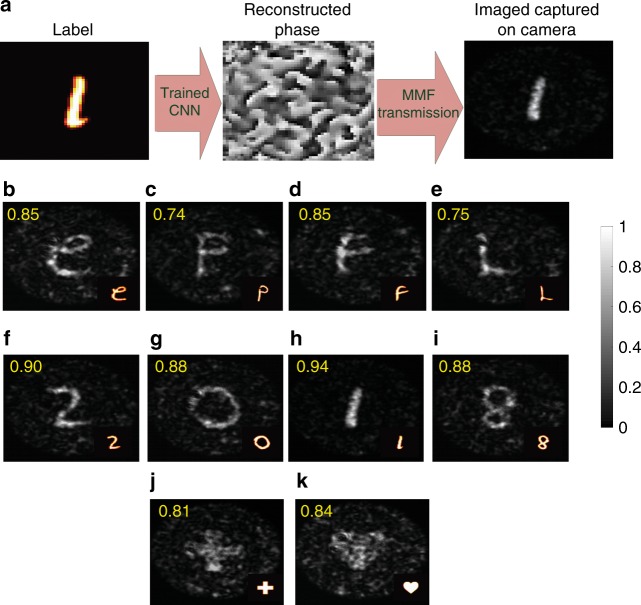
Experimental validation. **a** Schematic of the SLM phase inference by the CNN. The trained neural network reconstructs SLM phases from unseen images. The reconstructed phases are then sent through the MMF. Outputs of the fiber are then captured by the camera. The network, which is trained to regenerate input phases that output only the Latin alphabet, is also able to regenerate input phases belonging to other categories (transfer learning). Captured images of (**b**–**e**) Latin alphabets, (**f**–**i**) digits, (**j**) a cross and **k** a heart at the output of the fiber. The fidelity for each transmitted image with respect to the same transmitted image obtained using the transmission matrix of the system (sending ground truth SLM phases (labels), which are calculated by the inverse of the transmission matrix through the fiber, and capturing output images by the camera) is also shown

**Fig. 5:**
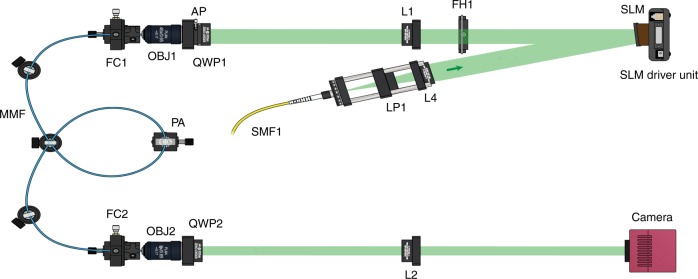
Optical setup. A schematic of the experimental setup for the transmission of light through the fiber. The pattern created by the SLM is imaged through the relay system (lens L1 and objective lens OBJ1) at the MMF input. An identical relay system (OBJ2 and L2) magnifies the image transmitted through the fiber and projects it on the camera plane

We used the measured transmission matrix and computed its inverse. The matrix inverse was then used to compute the SLM phase patterns that produce the amplitude images of the database of handwritten alphabets at the output. The VGG-net CNN is then trained with this set. Supplementary Figure S1 plots the MSE between the labels and the reconstructed SLM phase patterns produced by the trained CNN. As illustrated in the diagram of Fig. 4, the phase patterns generated by the network are then displayed on the SLM, transmitted through the fiber and captured by the camera using the optical setup in Fig. 5. Figure 4 depicts images of the Latin alphabet (b–e), digits (f–i), a cross, and a heart (j, k) captured by the camera at the output of the fiber.

## Discussion

We have shown that essentially, deep CNNs trained with a database of handwritten Latin alphabet letters were able to learn two types of highly nonlinear relations between the two-dimensional spatial output amplitude of an MMF and the two-dimensional spatial phase/amplitude at the input of the fiber. Interestingly, the performance difference between VGG-net and Res-net further confirms the presence of considerable nonlinearity in the learning system. We observe that a better performance is achieved with Res-net, which has a more complex architecture and thus is better suited for more complex problems.

### Amplitude-to-amplitude inversion

In the case where the inverse mapping is a conversion from output amplitude speckles to input amplitude patterns, the nonlinearity is due to the intensity-only detection at the distal end of the fiber, where the phase information is lost. Hence, the network is effectively learning to reconstruct the amplitude of the fiber input optical field *I*(*x*, *y*) from the amplitude of the fiber output complex optical field *E*(*xy*) such that $H\left( {\left| {E(x,y)} \right|} \right) = \left| {I(x,y)} \right|$, wherein *H* (.) is the feed-forward operator of the trained CNN used in this inverse problem.

In this manner, we showed that while the performances of the two CNNs, i.e., VGG-net and Res-net, are similar in terms of fidelity (~93% vs. ~96%), the training time of the latter is noticeably shorter (4 h 35 min vs. 1 h 20 min).

### Amplitude-to-phase inversion

When the network is trained to infer the input phases from the output amplitude speckles, another form of nonlinearity due to the exponential dependence of the phase is also introduced. Thus, in this scenario, the network is automatically learning to recover the phase *ϕ*(*x*, *y*) in an inverse mapping, in which the amplitude of the fiber output complex optical field, i.e., $\left| {E(x,y)} \right|$, is used as the input of the CNN feed-forward operator *H* (.):

$H\left( {\left| {E(x,y)} \right|} \right)$ = ∠*I*(*x*, *y*) = ∠*e*^*iϕ*(*x*, *y*)^ = *ϕ*(*x*, *y*), where ∠ is the argument operator. We observe that the training takes longer and converges to a higher MSE than that in the amplitude case. We attribute this partly to the fact that there is a high level of resemblance among the output amplitude speckle patterns when different handwritten Latin alphabet examples are shown, and partly because the network needs to learn a double nonlinearity. We showed that this problem of convergence rate could be significantly improved by using Res-net style networks. Not only does Res-net perform better in terms of training time but also it performs better in terms of fidelity. We achieved a fidelity of 79% for VGG-net and 88% for Res-net with training times of 9 h 0 min for VGG-net and 1 h 0 min for Res-net. Conspicuous differences in fidelity numbers further show the necessity of using a more complex architecture when dealing with highly nonlinear inverse problems.

### Transfer learning and image projection

Remarkably, we showed that our CNN is able to achieve transfer learning, i.e., reconstruct/transmit the desired patterns that did not belong to the class of images used to train the network. Specifically, we found that the VGG-net CNN was able to reconstruct the class of handwritten digits with an ~90% fidelity.

In a similar manner and in another experiment, we have shown that the network can be trained successfully with a different set of input–output images. In particular, we used a set of input plane waves at different angles filling the numerical aperture of the fiber and their corresponding speckle amplitude patterns at the fiber output (see the Supplementary Materials). In this case, very similar results in terms of image fidelity reconstruction were obtained (Supplementary Fig. S2). Interestingly, this network did not perform well in transfer learning (Supplementary Fig. S4). It is thus important to use a proper set of training examples. We posit that there could be other sets of images (than the handwritten Latin alphabet), which could work well with the MMF. Notably, the transmission matrix of the system is similarly built using a proper set of *complex* input–outputs using nontrivial interferometric approaches. The proposed CNN-based method, however, learns to relate the nonlinear amplitude-to-phase/amplitude inversion in the *real* domain. Thus, the network is effectively learning a subspace, instead of the complete space, which the matrix learns, by using a much simpler noninterferometric optical setup. Replicating the transmission matrix also brings advantages for image projection in MMFs. In this manner, we showed that our CNN could implement transfer learning for image projection, i.e., it can generate input phases that project arbitrary patterns at the distal end of the MMF.

## Materials and methods

### Experimental set-up

The optical setup for the transmission of light through the fiber is depicted in Fig. 5. The system here is a step-index (length = 0.75 m) MMF with a 50 µm diameter silica core and a numerical aperture of 0.22 (1055 number of fiber modes^33^). The inputs correspond to 2D phase patterns displayed on a phase only SLM, which are then demagnified on the MMF entrance facet by the 4 F system composed of lens L1 and OBJ1. The MMF output facet is imaged onto a camera.

The light source is a continuous wave source at 532 nm with a power of 100 mW, which is attenuated with a variable attenuator to deliver only 1 mW for the acquisition of the images. The light source is coupled into a single mode fiber. The light beam coming out of the SMF1 (object beam) is filtered by the polarizer LP1, collimated by the lens L4 and directed on the SLM, which can spatially modulate the impinging light. The pattern created by the SLM is imaged through the relay system (lens L1 and objective lens OBJ1) at the MMF input. The quarter wave plate (QWP1) before the fiber input changes the polarization from linear to circular (this polarization is better preserved in step-index fibers^34^). Then, light travels through the fiber and at the output, an identical relay system (OBJ2 and L2) magnifies the image of the output and projects it on the camera plane (the QWP2 converts the circular polarization back to linear).

### Neural network architecture

The problem of inferring the input phases/amplitudes from the output amplitude speckle patterns can be well studied in the framework of learning-based approaches, in which one seeks to solve an inverse problem based on available examples that are related to each other via an operator representing the physical system, here the fiber.

Let *I*(*x,y*) and *E*(*x,y*) denote the complex optical fields at the input and output of the fiber, respectively. Our objective is to find the input fields, either the amplitude or the phase of *I*(*x,y*), from the amplitudes of the output optical fields, i.e., $\left| {E(x,y)} \right|$. Mathematically, the objective function for amplitude-to-amplitude inverse problem reads as^24^

1 $$\theta ^o = {\mathrm{arg}}\,{\mathrm{min}}\,f\left[ {H\left( {\theta ,\left| {E(x,y)} \right|} \right),\left| {I(x,y)} \right|} \right]$$

in which *H* is the feed-forward operator and *f* is the cost function. In the case of an amplitude-to-phase conversion, $\left| {I(x,y)} \right|$ is replaced with ∠$I(x,y)$. The feed-forward operator *H* is basically the CNN, comprising linear multiplicative and additive weights and biases as well as nonlinear units. Learnable parameters of the network are denoted by *θ*. The ultimate goal is to find optimal values of these parameters, denoted by *θ*°, in a way that the operator *H* (which in principle is representing light back-propagation in the MMF) minimizes the cost function.

**Fig. 6:**
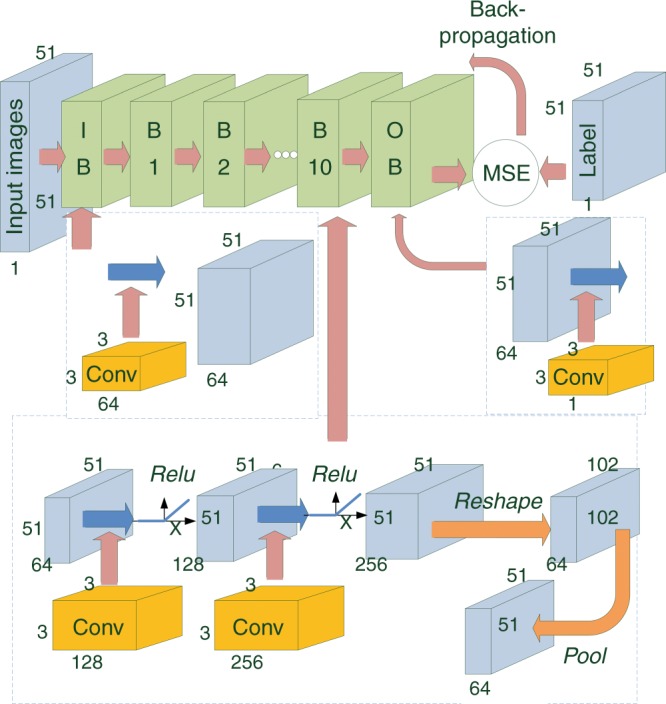
Neural network architecture. Detailed schematic of the CNN used for training and testing. IB (Input Block), OB (Output Block), B*i* (Block *i*, where *i* = 1, 2,…, 10), Pool (Max-pooling), Reshape (reshaping unit). The input block maps the input images via 64 convolutional filters. Each middle block (B1–B10) contains two convolution layers followed by a reshape and max-pooling layer, which together downsample the widths and heights of the images by a factor of two. A rectified linear unit (Relu) transform is placed after each convolution unit in the hidden layers. The images are then mapped to the output channel via the convolution filters in the output block. The MSE between the labels and the processed images is then calculated and back propagated to the network to update the learnable variables

The architecture of the VGG-net CNN (feed-forward operator *H*) is schematically shown in Fig. 6 (the architecture of the Res-net CNN is discussed in detail in the Supplementary Materials). This architecture consists of 12 blocks, in which the first and the last are the input and output units that are responsible to encode and decode data to the network, respectively. The input block maps the grayscale input images (one channel) to 64 channels (stack of processed images) via a trainable convolutional unit. The middle 10 blocks constitute the 20 hidden layers of the neural network, wherein each block is formed by two convolutional entities that are individually followed by an element of the rectified linear unit^35^ (*RELU*) with a mapping functionality *RELU*(*x*)=max(0,*x*). The convolutional layers within each block take the convolution of the stack of input feature map $X_k^q$ using weights $W_k^q$ and biases $B_k^q$ complying with the formula $X_{k + 1}^q = Conv_{W_k^q}(X_k^q) + B_k^q$, where the subscript *k* indicates the layer number and the superscript *q*={1,2} corresponds to the first and second convolution operations in each and every block. Mathematically, the convolution operator outputs the input feature maps $X_k^q$ as^27^:

2 $${\bar X_{k + 1,r}^q(i,j) = \mathop {\sum}\limits_p {\mathop {\sum}\limits_{n_1 = 0}^2 {\mathop {\sum}\limits_{n{}_2 = 0}^2 {\bar X_{k,p}^q(i + n_1,j + n_2)\bar W_{k,r,p}^q(n_1,n_2)} } }}$$

where *i* and *j* are the pixel indices of the feature maps and $\bar X_{k,p}^q$ denotes the *p-*th input feature map of the stack $X_k^q$ inside the *k*-th layer and before the *q*-th convolution unit in that layer. Likewise, $\bar W_{k,r,p}^q$ indicates a kernel of weights belonging to the *k*-th layer and the *q*-th convolution, which is multiplied to the *p*-th input feature map and outputs the *r*-th feature map in the next layer. We have used only 3 × 3 convolution kernels and thus, the pixel indices of the kernels, i.e., *n*_1_ and *n*_2_, take values from 0 to 2.

At the output of each block, an additional *Max-pooling*^36^ unit is considered. *Max-pooling* units help to avoid overfitting and therefore are essential parts of the network. These units decrease the widths and heights of the images passing through them by a factor of two. To keep the dimension of the images constant throughout the network, additional *Reshaping* units are placed just before the *Max-pooling* units. Supplementary Figure S7 depicts the detailed schematic of the *Reshaping* elements. These units reorder the stack of 256 *M*x*M* images into 64 2*M*x2*M* images. The *Max-pooling* units then downsample the 2*M*x2*M* images back to *M*x*M* images.

The final block is made of a convolutional layer that simply decodes back the images from 64 channels to the original single-channel grayscale images. The architecture of the network in this work follows the standard block-structure (*conv*-*RELU)*→(*conv*-*RELU)*→*MaxPool*, which is adopted from VGG-Nets (VGG19) and customized by adding the *Reshaping* units before the *Max-pooling* units. Increasing the number of layers or the number of channels (very deep and wide architectures) adds to the complexity of the network. It is a well-known fact from the generalization theory in machine learning that more complex networks require more training data to overcome overfitting. On the other hand, the ability of the network to generalize degrades when shallow/thin networks are used. Therefore, the number of hidden layers, herein 20 for the VGG-net architecture, as well as the number of output (input) channels in the input (output) block, i.e., 64 channels, is empirically chosen based on a trade-off among the network’s complexity, the number of training data, and the level of accuracy desired to obtain the optimal results. Additionally, more complex networks require processing units that are able to do computationally intensive calculations more rapidly. Therefore, the complexity of the network is also balanced here with the available hardware power as well as the image output time.

Once the images are obtained in the final layer of the CNN (feed-forward step), they are compared with their corresponding labels in a MSE sense. The MSE in this comparison that is used for updating the learnable parameters in the stochastic gradient descent^37^ algorithm reads as follows:

3 $$MSE(\theta ) = \frac{1}{{N \times M \times M}}\mathop {\sum}\limits_{l = 1}^N {\mathop {\sum}\limits_{i = 1}^M {\mathop {\sum}\limits_{j = 1}^M {|Y_{i,j}^{R_l}(\theta ) - Y_{i,j}^{L_l}|^2} } }$$

where *θ* is the CNN’s set parameters (including weights and biases), *i* and *j* are the indices of the neural network reconstructed image $Y^{R_l}$ and the label image $Y^{L_l}$ belonging to the *l*-th image pairs, where *l* and *N* are the samples’ mini-batch index and size, respectively, and *M* is the width and height of the images. Once the stochastic MSE function in Equation (3) is calculated, it is optimized using the adaptive moment estimation optimization (ADAM) algorithm^37^. To obtain accurate results within a reasonable time, we empirically choose a learning rate parameter of 10^−4^ in the optimization algorithm and a mini-batch size of 64 (to expedite training, we choose a higher learning rate (10^−3^) for the CNN that is used for experimental validation. The batch size in this case is 20). Details of the technical implementation including the platform for writing the back-propagation algorithm as well as the specifications of the processing units can be found in the Supplementary Materials.

### Data preparation

Throughout the paper, the field amplitude at the output of the MMF, i.e., $\left| {E(x,y)} \right|$, is used as the input of the neural network and its corresponding phase or amplitude pattern at the proximal end of the fiber, i.e., ∠$I(x,y)$ or $\left| {I(x,y)} \right|$, is used as the labels to the network. We use the transmission matrix of the system, referred to as *T* hereafter, which is obtained via the optical setup schematically depicted in Supplementary Fig. S5, to generate the input/label pairs for the training and test datasets. The procedure to obtain the matrix is further explained in the Supplementary Materials. The transmission matrix method has been shown to be a robust method to accurately characterize the transmission of light through MMFs and thus provide a convenient method to generate the datasets.

In a more detailed fashion, the dataset for training the network used with the Latin alphabet is obtained as follows. The transmission matrix is utilized to compute the output amplitude speckle patterns of the input amplitude/phase Latin alphabet adopted from Ref. ^32^:

4 $$E(x,y) = F^{ - 1}\left[ {T.F\left[ {I(x,y)} \right]\,} \right]$$

where *F* and *F*^−1^ denote the Fourier and inverse Fourier transforms, respectively. Accordingly, pairs ${\kern 1pt} \left\{ {\left| {E(x,y)} \right|,\left| {I(x,y)} \right|} \right\}$ and pairs {${\kern 1pt} \left| {E(x,y)} \right|$, ∠*I*(*x*, *y*)} constitute the data used for the amplitude-to-amplitude and amplitude-to-phase inversions, respectively.

Likewise, the data set for training the network used for experimental verification are obtained as follows. The inverse of the transmission matrix is used to generate the SLM phases that experimentally output images of the Latin alphabet, i.e., phases that produce specific alphabet images if illuminated on the fiber via the SLM:

5 $$\angle I(x,y) = {\mathrm{arg}}\,F^{ - 1}\left[ {T^{ - 1}.F\left[ {E(x,y)} \right]} \right]$$

## Electronic supplementary material

Supplementary-Information

Moving Donuts

Running Dog
