# Supplementary material for: Multimode optical fiber transmission with a deep learning network
Source: Light Sci Appl. 2018 Oct 3;7:69. doi: 10.1038/s41377-018-0074-1 (PMC6168552; doi:10.1038/s41377-018-0074-1)
Supplement: Supplementary file 1 — Supplementary-Information [file 41377_2018_74_MOESM1_ESM.docx]

Supplementary Information for

Multimode optical fiber transmission with a deep learning network

Babak Rahmani,^1,*^ Damien Loterie,^1^ Georgia Konstantinou,^1^ Demetri Psaltis,^2^ Christophe Moser^1^

*^1^Ecole Polytechnique Fédérale de Lausanne,* *Laboratory of Applied Photonics Devices,* *CH-1015 Lausanne, Switzerland*

*^2^Ecole Polytechnique Fédérale de Lausanne, Laboratory of Optics, CH-1015 Lausanne, Switzerland*

**Corresponding author:* [*babak.rahmani@epfl.ch*](mailto:babak.rahmani@epfl.ch)

**Performance of the CNN used for experimental validation**

Performance of the CNN used for experimental validation is discussed here. Figure S1 plots (a) the MSE between (b) the label (original phase pattern obtained by the inverse of the transmission matrix) and (c) the reconstructed phase pattern (obtained by the CNN) for letter “e” in Fig. 4 (b) in the main manuscript.

**Amplitude-to-phase conversion using the CNN trained with phases of plane waves**

We have demonstrated that the network can be also trained successfully with a set of input plane waves at different angles filling the numerical aperture of the fiber and their corresponding speckle amplitude pattern at the fiber output. The dataset for training the CNN is obtained as follows: 100 phase angles which correspond to plane waves illuminating the bottom of the numerical aperture of the fiber are chosen (the number of all possible input plane waves filling the entire numerical aperture of the fiber is 1961). The speckle pattern obtained upon transmission of each phase angles through the fiber is measured 50 times. This is equivalent to measuring the transmission matrix, 50 times, and then computing the output speckle pattern (using the transmission matrices) corresponding to each phase angle for all 50 matrix measurements. It should be mentioned that an interval of 10 minutes is considered between each transmission matrix measurements. In this way, 50 sets of data, each containing 100 input-output images of size $51\times51$ pixels are obtained. Out of 50 sets each containing 100 input-output images, 40 sets are randomly selected to be used as our training data, while the remaining 10 sets are used for validation. Therefore, in total, 4000 (40×100) image pairs are used for training and 1000 (10×100) pairs for validation. It should be noted that the output speckles corresponding to different measurements are different from one another due to various random changes such as thermal drift, fiber misalignment, etc. occurring as time elapses. The aim behind collecting data in this way is to show that the network can still determine the input phase angles although the speckle patterns undergo changes due to external features.

Figure S2 plots (a) the MSE as well as (b) 2D-correlation between (c) the labels and (d) the reconstructed phase patterns versus the number of iterations for both the training and the validation dataset. The training time of the neural network in this case is ~1hr, 20min. We observe a correlation of 98.25% between the labels and the reconstructed phases on the validation set.

To show that the network can still learn the non-linear amplitude-to-phase inversion even if the amplitude images have some artifacts within them (fringes due to holographic transmission), the neural network was also trained with the raw hologram amplitudes (instead of the field amplitude) obtained at the output of the MMF. From a total of 50 sets each containing 100 angles illuminating the bottom of the numerical aperture of the fiber (similar to the previous case), 40 sets are randomly selected for training and 10 sets for validation. Hence, 4000 (40×100) image pairs are used for training and 1000 (10×100) pairs for validation. It should be noted that the output raw holograms are obtained by sequentially sending the plane waves and capturing the holograms on the camera. Additionally, raw holograms are bicubically resized from 576x576 to 51x51 to feed the network. Figure S3 depicts (a) the MSE as well as (b) 2D-correlation between the labels and the reconstructed input phase patterns versus the number of iterations for both the training and the validation dataset. The training time of the neural networks in this case is ~2hr, 30min. The time for training the network when raw holograms are fed is higher than the time for training with the optical field amplitudes. It is expected since for the case of raw holograms, there is the additional phase information between the propagating modes as well as some artifacts due to the holographic measurements which have to be learned. Nevertheless, the performance of the network to learn the inverse propagation (amplitude-to-phase relation of the MMF system) is very similar in terms of correlation (98.25% vs. 97.28%).

**Transfer learning using the CNN trained with phases of plane waves**

For showing that the network cannot do transfer learning when it is trained with phases of plane waves, one may plausibly expect that this approach could possibly work if the CNN is trained to recognize all the possible input plane waves filling the numerical aperture of the fiber which is 1961 in total. Therefore, we again train the CNN with 1961×10 pairs of images (corresponding to measuring the transmission matrix 10 times and computing the output speckle patterns for all 1961 illumination angles 10 times) of which, 8 sets of data each containing 1961 patterns (1961×8) are used for training and 2 sets for validation (1961×2). Once the CNN is trained, the ability of the network in transfer learning can be evaluated. For this, we choose a MMF input image (a cross picture) for which the corresponding output speckle does not belong to the category produced by the plane wave phase patterns. We observe that the CNN generates a phase pattern not similar to the desired input (Fig. S4). Although the network has learned the amplitude-to-phase relationship with patterns representing the Fourier basis, it is not capable to effectively learn the generalized fiber transmission.

**Optical setup and the procedure for obtaining the transmission matrix of the multimode fiber**

Figure S5 plots the optical setup for obtaining the transmission matrix of the Multimode fiber (MMF). It should be emphasized that the holographic system is used to only measure the transmission matrix which itself is used as gold standard against which we test the performance of the CNN of the system and does not play any role in the neural network training procedure.

The system here is again a step-index (length=0.75 m) MMF with 50um diameter silica core and numerical aperture 0.22. The inputs correspond to 2D phase patterns displayed on a phase only spatial light modulator (SLM) which are then demagnified on the MMF entrance facet by the 4F system composed of lens L1 and OBJ1. The MMF output facet is imaged onto a camera and which interferes with a coherent beam (fiber split to SMF2) to yield an off-axis hologram. The light source is similarly a continuous wave source at 532nm and power 100mW. However, it is attenuated with a variable attenuator and only 1mW is used for the acquisition of the images. The light source is coupled into a single mode fiber. A fiber coupler is used for obtaining two different ports (SMF1 and SMF2). The light beam which is coming out of the SMF1 (object beam), is filtered by the polarizer LP1, collimated by the lens L4 and directed on the SLM. The pattern created by the SLM is imaged through the relay system (lens L1 and objective lens OBJ1) at the MMF input. The quarter wave plate (QWP1) before the fiber input changes the polarization from linear to circular. Then it travels through the fiber and at the output an identical relay system (OBJ2 and L2) magnifies the image of the output and projects it on the camera plane (the QWP2 converts the circular polarization back to linear). The light beam originating from the SMF2 (reference beam) is filtered by the polarizer LP2 and collimated by the L5. The mirrors KM1, KM2 and the beam splitter BS are used to adjust the off-axis angle of the reference arm. The BS is used to combine both beams in order to interfere on the camera plane. The FC1 and FC2 are fiber clamps and the FH3 is a filter holder.

To measure the transmission matrix of the system, a sequence of plane wave phase patterns $\Phi_{n}(x,y)$, where index $n$ represents the numbering of the pattern in the sequence and $x,y$denote the Cartesian variables, is first created and then loaded onto the SLM which is then imaged onto the fiber input facet. The speckle patterns formed due to the modal interference inside the fiber are collected at its output facet by the camera as raw off axis holograms. A preprocessing step is carried out to obtain the optical field amplitudes$O_{n}(x,y)$. First, the raw holograms are Fourier transformed and then all areas except the holographic real-order are masked out in the Fourier domain. By taking the inverse Fourier transform, the field amplitude is reconstructed. This process is illustrated in Fig. S6. Hence, by cropping the Fourier-domain image around the holographic real-order, the size of the images are decreased from 576x576 to 51x51 pixels.

**Neural network technical implementation details**

The program for training the CNN was written using Tensorflow, a Python-based open-source library developed by Google for implementing neural networks. The Tensorflow version used here is 1.5 running on Python version 3.5.4. The machine used to run the simulations is a windows-based computer with Intel (R) Xeon (R) CPU E5-2609 v3@ 1.9GHz and 32 GB of RAM. We used NVIDIA Quadro M4000 graphic processing unit (GPU) as the platform for running Tensorflow.

**Details of the Reshaping units used in the architecture of the convolutional neural network**

Maxpooling^1^ units divide the heights and widths of images passing through them by a factor of 2. Thus, to keep the dimensions of the images constant, additional Reshaping units^2^ are added to the network before each Maxpooling. Detailed schematic of their operation is illustrated in Fig. S7. These units re-order the stack of 256 images into 64 images. The Maxpooling units down-sample the images back to images afterwards.

**Residual Network (Res-net)**

The architecture of the Res-net CNN is schematically shown in Fig. S8. It consists of 9 blocks; of which, the first and the last are made of a single convolution layer followed by a non-linear rectified linear unit (*RELU*) which, respectively, takes one channel (64 channel) gray-scale input images (output images) and maps them to 64 channels (1 channel) of stack of images. A batch-normalization^3^ unit is considered after all convolutional layers throughout the network and also one at the very beginning of our architecture. We only have used 3×3-kernel convolutional layers. The 6 blocks in the middle of the network, known hereafter as residual blocks 1 to 6, are made of two convolution layers that are both followed by *RELUs*. The architecture of the network is based on Residual neural networks^4^; hence, a skip connection is added to the output of the second convolution layer at the end of each and every residual block and before the *RELU*. In the case where dimensions of feature maps after convolutional layers increase (residual block 3), extra zero entries are added to match dimensions. *Max-pooling* units with sizes 2×2 and strides of 2 are considered after residual blocks 1 to 4, In a similar fashion, up-sampling units are added after residual blocks 5, 6 and block 7. Note that block 7 does not have any skip connections and therefore, is not a residual bock.

We have used the proposed Res-net for the task of output speckle amplitude to input amplitude and output speckle amplitude to input phase conversions similarly to what we did using VGG-net CNN. The learning rate is also similar to the learning rate of VGG-net (10^-4^) to ensure a fair comparison in terms of the convergence rate and training time for the two architectures. Figure S9 plots the 2D-correlation for amplitude-to-amplitude (a) and amplitude-to-phase (b) inversions. Comparison between the training time and fidelity number between the two structures reveals the superiority of Residual architectures for these tasks (see Results section in the main manuscript).

**Supplementary Movie 1**

A “Moving Donuts” animation is prepared. The donuts are positioned at 30 different spots. Images of speckle patterns associated with donut images are fed to the *amplitude-to-amplitude* network trained on Latin alphabets images. The Movie contains the CNN-reconstructed amplitude images (right), the speckle patterns (middle) and the labels (left).

**Supplementary Movie 2**

An animation of a “Running Dog” containing 8 frames is reproduced via the *amplitude-to-amplitude* network trained on Latin alphabets images. The Movie contains the CNN-reconstructed amplitude images (right), the speckle patterns (middle) and the labels (left).

**Fig. S1.** The performance of the neural network used for experimental validation. Calculated (a) MSE between the (b) label and (c) the corresponding reconstructed phase pattern of the letter “e” in Fig. 4 (b) in the main manuscript.

**Fig. S2.** The performance of the neural network on the training and validation dataset. Calculated (a) MSE, (b) 2D-correlation between (c) the labels (original phase patterns) and (d) corresponding reconstructed phase patterns by the CNN when the field amplitude of the MMF output patterns are fed to the network as input.

**Fig. S3.** The performance of the neural network on the training and validation dataset. Calculated (a) MSE, (b) 2D-correlation between the labels and the reconstructed input phase patterns when raw holograms are fed to the CNN.

**Fig. S4.** a) Input phase image on the SLM from a category of images not used to train the CNN. (b) Computed phase image on the SLM by the CNN. The network is not able to reconstruct the proper pattern.

| 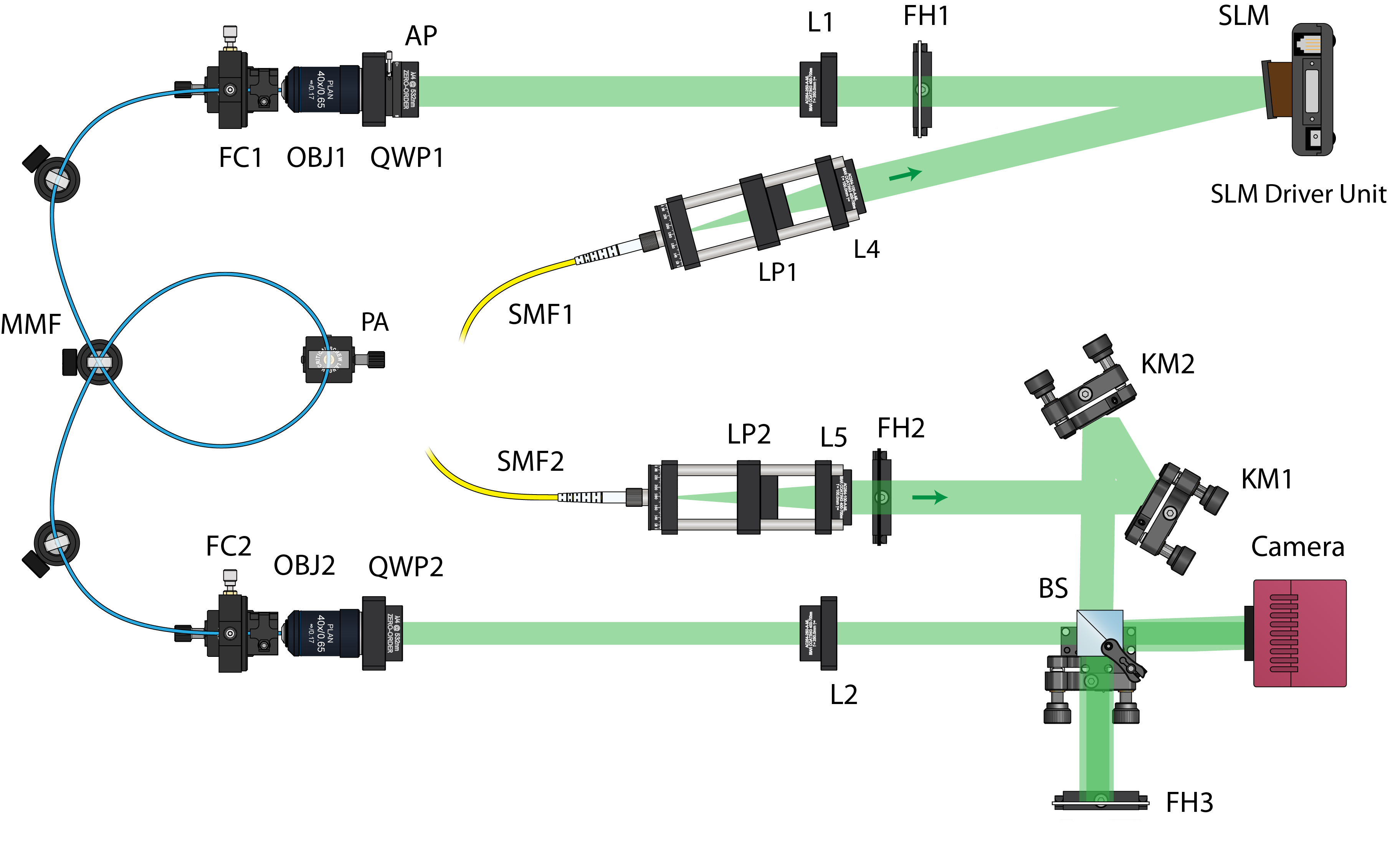 |
| --- |
| **Fig. S5.** A schematic of the experimental setup for measuring the transmission matrix. |

**Fig. S6.** Object optical field amplitudes reconstruction steps. Raw holograms are first Fourier transformed and masked with proper filter. Inverse Fourier transform gives the reconstructed object wave. (a) Raw off-axis hologram at the output of the MMF as recorded by the camera. (b) Fourier transform of the hologram shown in (a), represented here with a logarithmic amplitude coloring. (c) Amplitude of the inverse Fourier transform of masked out area (dashed circle in (b)).

**Fig. S7.** Detail schematic of the Reshaping unit’s operation.

**Fig. S8.** Detail schematic of the Res-net architecture.

**Fig. S9.** Calculated 2D-correlation for train/validation datasets for amplitude-to-amplitude (a) and amplitude-to-phase (b) inversions. (c) Examples of the output amplitude speckle patterns and the reconstructed fiber input amplitude/phase patterns produced via the CNN. The fidelity number for each reconstructed image with respect to its corresponding label is shown.

**References**

1. Simonyan, K. & Zisserman, A. Very deep convolutional networks for large-scale image recognition. *arXiv preprint arXiv:1409.1556* (2014).
2. Rivenson, Y. *et al.* Deep learning microscopy. *Optica, OPTICA* **4,** 1437–1443 (2017).
3. Ioffe, S., & Szegedy, C. Batch normalization: Accelerating deep network training by reducing internal covariate shift. *arXiv preprint arXiv*:1502.03167 (2015).
4. He, K., Zhang, X., Ren, S., & Sun, J. Deep residual learning for image recognition. In *Proceedings of the IEEE conference on computer vision and pattern recognition* (pp. 770-778) (2016).
