# Supplementary figures and images for: Multimode optical fiber transmission with a deep learning network
Source: Light Sci Appl. 2018 Oct 3;7:69. doi: 10.1038/s41377-018-0074-1 (PMC6168552; doi:10.1038/s41377-018-0074-1)

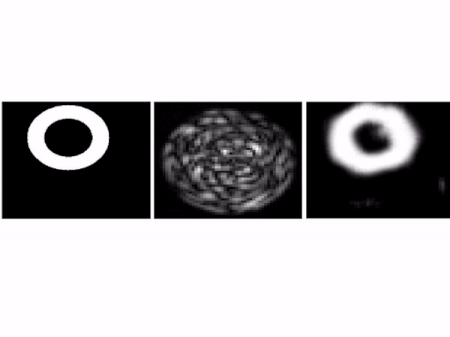

Supplement: Supplementary file 2 — Moving Donuts [file 41377_2018_74_MOESM2_ESM.gif]

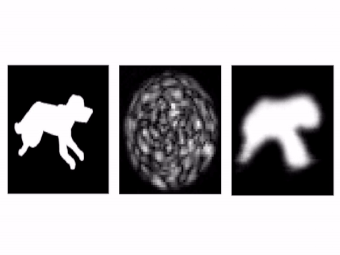

Supplement: Supplementary file 3 — Running Dog [file 41377_2018_74_MOESM3_ESM.gif]
